# Cardiovascular autonomic modulation differences between moderate-intensity continuous and high-intensity interval aerobic training in women with PCOS: A randomized trial

**DOI:** 10.3389/fendo.2022.1024844

**Published:** 2022-11-25

**Authors:** Stella V. Philbois, Victor B. Ribeiro, Jens Tank, Rosana Maria dos Reis, Darius A. Gerlach, Hugo C. D. Souza

**Affiliations:** ^1^ Laboratory of Physiology and Cardiovascular Physiotherapy, Department of Science of Health, Ribeirão Preto Medical School, University of São Paulo, Ribeirão Preto, São Paulo, Brazil; ^2^ Department of Gynecology and Obstetrics, Ribeirão Preto Medical School, University of São Paulo, Ribeirão Preto, São Paulo, Brazil; ^3^ Department of Cardiovascular Aerospace Medicine, Institute of Aerospace Medicine, German Aerospace Center, Cologne, Germany

**Keywords:** aerobic physical training, cardiovascular autonomic control, heart rate variability, high-intensity interval training, polycystic ovary syndrome

## Abstract

**Background:**

Moderate-intensity continuous training (MICT) is strongly recommended for polycystic ovarian syndrome (PCOS) treatment. However, recent studies have suggested that high-intensity interval training (HIIT) would promote great benefits for cardiac autonomic control. Therefore, we investigated whether the benefits of HIIT related to cardiovascular autonomic control were greater than those of MICT in women with PCOS.

**Methods:**

Women with PCOS were randomly allocated through a blind draw into three groups: control, MICT, and HIIT. The control group did not undergo exercise, whereas those in the MICT and HIIT groups underwent 16 weeks of aerobic physical training. All groups were evaluated before and after the 16 weeks of intervention in the following aspects: quantification of serum lipids, testosterone, fasting insulin and blood glucose; physical fitness through cardiopulmonary testing; analysis of heart rate variability (HRV) by linear (time domain and frequency domain) and non-linear (symbolic analysis) methods, analysis of blood pressure variability (BPV) and spontaneous baroreflex sensitivity (BRS).

**Results:**

The final analysis, each group comprised 25 individuals. All groups had similar baseline parameters. After 16 weeks, intragroup comparison showed that the MICT and HIIT groups had a reduction in baseline heart rate (P < 0.001; P < 0.001, respectively) and testosterone levels P < 0.037; P < 0.012, respectively) associated with an increase in VO2_peak_ (MICT, P < 0.001; HIIT, P < 0.001). The MICT (P < 0.36) and HIIT (P < 0.17) groups also showed an increase in cardiac vagal modulation, however only observed in the non-linear analysis. The intergroup comparison showed no differences between the MICT and HIIT groups in any of the hormonal, metabolic and autonomic parameters evaluated, including testosterone, peak oxygen uptake (VO2_peak_), HRV, BPV and BRS.

**Conclusion:**

HIIT and MICT showed similar results for the different parameters evaluated. This suggests that both training protocols can be recommended for the treatment of PCOS. Brazilian Clinical Trials Registry (RBR-78qtwy).

## 1 Introduction

Polycystic ovary syndrome (PCOS) affects a large proportion of women of reproductive age. Studies have demonstrated a prevalence between 6% and 13%; however, it varies depending on the diagnostic criteria used (1–4). This high prevalence is concerning, since PCOS may lead to insulin resistance, obesity, and cardiovascular autonomic changes, predisposing the development of diabetes mellitus type 2 and cardiovascular diseases (CVDs) (2, 5, 6).

Therapeutic interventions that target the prevention of comorbidities resulting from PCOS include regular physical exercise (3, 4, 7). Some clinical studies demonstrate the long-term effects of moderate-intensity continuous aerobic training (MICT) and its beneficial effects on adjustments in the regulation of hemodynamic, metabolic, and cardiovascular autonomic control, especially insulin sensitivity and cardiac modulation of heart rate variability (HRV) (8–10).

High-intensity interval training (HIIT) is another aerobic physical training protocol that has been used to optimize therapeutic benefits, mainly in patients with different CVDs. Some studies have shown that HIIT promotes greater benefits for cardiorespiratory fitness, as well as hemodynamic and anthropometric parameters, compared to MICT (11–13). However, these therapeutic benefits are still controversial, especially those related to cardiovascular autonomic control (13–18).

Although aerobic physical exercise is well known for improving hemodynamic, metabolic, and cardiovascular autonomic control in people with different diseases, there are limited studies in the literature regarding the effects of physical training on cardiovascular autonomic modulation in women with PCOS. We found few studies, although they did not present an analysis of cardiovascular autonomic control (19–22). Thus, there is a great need to find others training methods that would promote great therapeutic benefits for patients with PCOS. The current study aimed to compare the effects of HIIT and MICT on cardiovascular autonomic control in women with PCOS evaluated using HRV, blood pressure variability (BPV), and baroreflex sensitivity (BRS).

## 2 Materials and methods

### 2.1 Sampling

**Figure 1:**
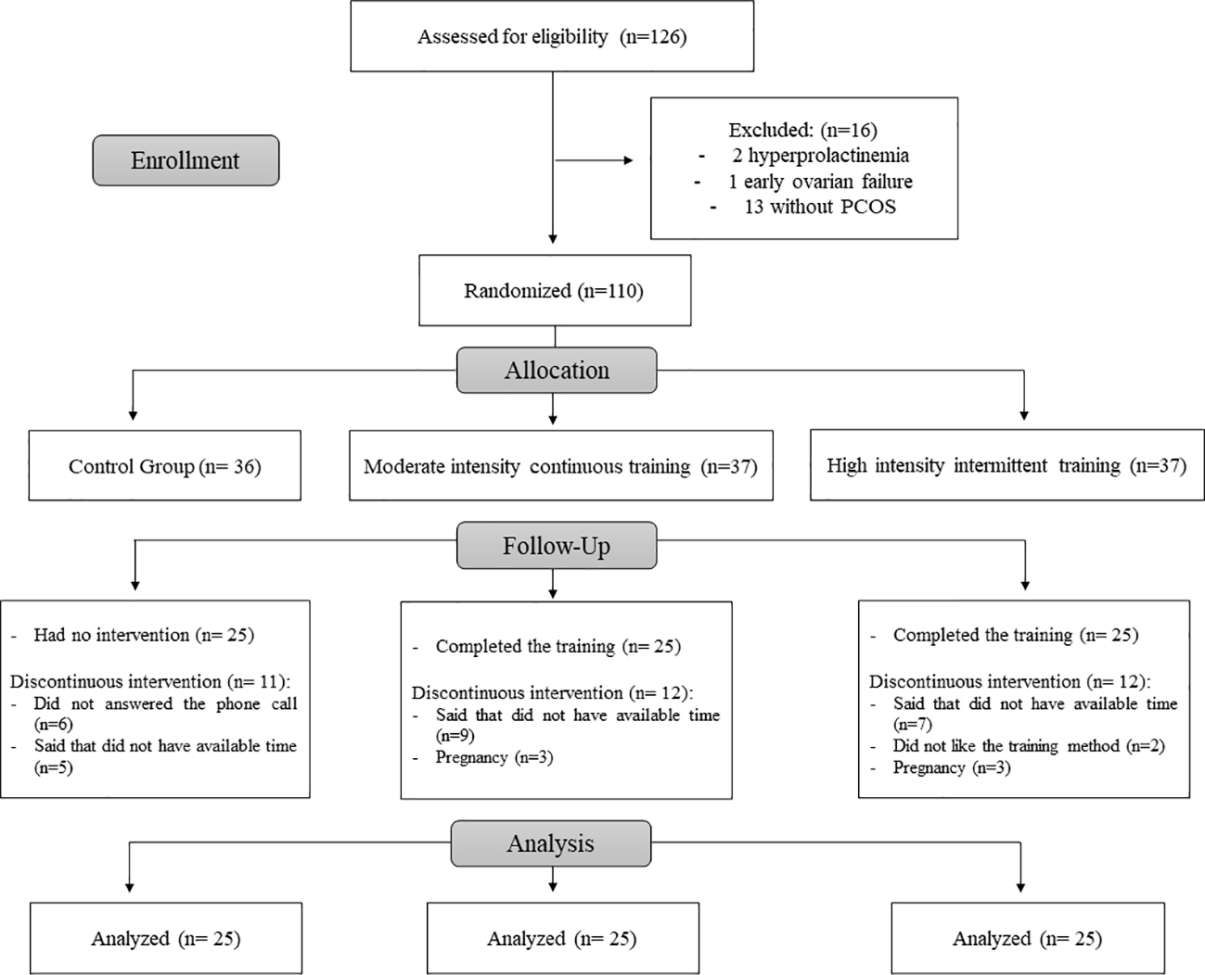
A flow diagram of the study.

This was a randomized clinical trial. In total, 126 participants aged between 18 and 39 years were enrolled, and 110 were included (**Figure 1**). All patients were screened at the Gynecology and Obstetrics Clinic of the Ribeirão Preto Medical School’s Hospital (HCFMRP/USP). The patients were diagnosed with PCOS according to the Rotterdam Consensus criteria (23). In the end, 75 participants completed the experimental protocol (**Figure 1**). The participants were randomly allocated to three different experimental groups: control (N = 25), without any exercise training; MICT group (N =25), and HIIT group (N = 25), and the study lasted for 16 weeks. Randomization was performed through a blind draw using numbers one to three, wherein each number corresponded to a specific group. The exclusion criteria were a history of a) smoking, b) cognitive disturbances, c) pregnancy, d) musculoskeletal disorders, e) CVDs, and f) the use of any medication, including contraceptives. The study was approved by the Ethics Committee of the Ribeirão Preto Medical School’s Hospital (Protocol number 845.830/2014), the scientific and legal aspects were disclosed to the participants, and all participants signed a free and informed consent form, agreeing to participate. The authors confirm that all ongoing and related trials for this intervention were registered with the Brazilian Clinical Trials Registry (RBR-78qtwy).

### 2.2 Protocols

Data were collected in the morning during two laboratory visits between 07:00 AM and 10:00 AM, with a 48-hour interval between visits. Data were collected during the follicular phase for women with regular ovulatory cycles and at any time for those with irregular ovulatory cycles. The first assessment included anthropometric measurements and blood collection performed in the laboratory of the HCFMRP/USP.

The second assessment was performed at the Laboratory of Exercise Physiology and Cardiovascular Physiotherapy of Ribeirão Preto Medical School. During the second visit, the following protocols were completed: anthropometric parameters, cardiovascular autonomic analysis, and cardiorespiratory function test. Each visit lasted approximately two hours.

All participants were asked to avoid exercise and consumption of alcoholic beverages and maintain their usual diet for 48 h prior to the assessments. They were also advised to sleep for at least 7 or 8 h the night before the visits.

#### 2.2.1 Laboratory tests

Blood samples (3.5 ml, BD Vacutainer^®^ EDTA - Becton, Dickin, and Company, Franklin Lakes, NJ, USA) were used to analyze fasting glycemia hexokinase-UV), insulin levels (chemiluminescence immunoassay), triglyceride levels (dehydrogenase), and total cholesterol and fraction levels (esterase-oxidase). All participants were asked to fast for 12 h prior to the assessments. Insulin resistance was assessed using the homeostasis model assessment-insulin resistance index (HOMA-IR) (24).

#### 2.2.2 Anthropometric parameters

Data on body weight and height were obtained using an analog scale with an altimeter (Welmy), while the body mass index (BMI) values were calculated using weight and height expressed as kg/m^2^.

#### 2.2.3 Cardiorespiratory function test

Peak oxygen uptake (VO2peak) was assessed by a submaximal exercise test conducted on a treadmill (Super ATL Millenium^®^, Inbramed/Inbrasport, Brazil) using the modified Bruce protocol. Heart rate (HR) was continuously monitored by electrocardiography with one lead (CM5). The analysis of exhaled gases (VO2 and VCO2) was performed using a metabolic device (PowerLab/8M, ADInstruments, Bella Vista, Australia).

#### 2.2.4 Hemodynamic assessment

Data on systolic blood pressure (SBP), diastolic blood pressure (DBP), and mean blood pressure (MBP) were obtained using digital plethysmography recording equipment (Finometer Pro, Finapres Medical System, Amsterdam, Netherlands). HR data were obtained using an electrocardiographic (ECG) digital recorder through the CM5 lead (ML866 PowerLab).

#### 2.2.5 HRV, BPV, and spontaneous BRS analysis

HRV data were obtained using the RR intervals (RRi) from the ECG records at a sampling frequency of 2000 Hz. The BPV values were obtained from the SBP data recorded beat-to-beat using the Finometer, with a cuff positioned on the middle finger of the right upper limb. The data interface to the microcomputer was performed using a PowerLab4/35 device (ADInstruments),. The data were recorded and stored (Software LabChart 8.0, ADInstruments), for further analysis. The participants were instructed to remain in the supine position for approximately 10 min to stabilize the cardiovascular parameters. After this period, the ECG signals and arterial pulse pressure were recorded simultaneously for another 10 min. The temperature (22°C) and ambient lighting were controlled, and the sessions were performed in a noise-free environment.

The HRV and BPV analyses were performed using custom computer software (CardioSeries v2.4, http://sites.google.com/site/cardioseries). The HRV was assessed by the following three methods: time domain (standard deviation [SD] and the root mean square of successive normal sinus RR-interval [RMSSD]) (25), frequency domain (spectral analysis, fast Fourier transform [FFT]) (25, 26), and nonlinear analysis (symbolic analysis) (27, 28). The BPV was assessed only by frequency domain (spectral analysis) (26). BRS was assessed in the time domain using the sequence technique (29, 30). The computer software CardioSeries v2.4 was also used as previously described (26).

#### 2.2.6 Training protocol

The training sessions were conducted at the Laboratory of Exercise Physiology and Cardiovascular Physiotherapy of the Ribeirão Preto Medical School. After the initial evaluation, both MICT and HIIT participants were submitted to their respective training protocols using a motorized treadmill. These sessions were supervised and monitored three times per week for a total of 16 weeks. MICT used the equivalent intensity of HR corresponding to the sum of HR at rest and 70%–80% of reserve HR, obtaining the following equation: HR recorded at peak cardiopulmonary test – HR at rest. The MICT training sessions had a duration of 1 hour, divided into three phases as follows: 5-min warm-up using intensity lower than the target HR training range (50%–65% of reserve HR), 50-min training using the HR target (70%–80% of reserve HR), and 5-min cool-down, using intensity lower than the training HR (40%–50% of reserve HR).

In contrast, the HIIT session was as follows: 5-min warm-up using intan ensdensitywer than the target HR training range (50%–65% of reserve HR); for the main phase of HIIT, the intensity used for a total of 2-min corresponded to the sum of HR at rest and 85%–90% of the reserve HR, altering with 3-min of intensity corresponding to the sum of HR at rest and 65%–70% of the reserve HR; and the last stage consisted of 5-min cool-down, using intensity lower than the training HR (40%–50% of reserve HR). The two aerobic exercise training protocols, HIIT and MICT, had similar total HR numbers. Every day of physical training, the HR of the participants was beat-by-beat monitored and recorded (Polar RS810). Stored records were quantified for necessary adjustments for the next physical training session. While MICT had a total training duration of 50 min, HIIT had a total duration of 35–45 min. Prior to both training models, during the first 2 weeks of the study, an adaptation period was provided to the participants. All participants underwent sessions of 20–30 min for familiarization and adaptation to the treadmill protocol. The intensity used was equivalent to the sum of HR at rest and 50%–60% of the reserve HR. In addition, if the participant did not have an adherence above 85%, their data were not included in the analysis.

### 2.3 Statistical analysis

The sample size calculation was performed using the Sigma-Plot^®^, version 11.0 software (Systat Software Inc., San Jose, CA, USA) with a confidence level of 95% and power of 80% using the LF and HF variables in normalized units, with a standard deviation of 15. We initially carried out an exploratory analysis of data through measures of central position and dispersion. A mixed effects linear regression model was adjusted to verify the effect of time and group variables in relation to the outcomes of interest. This model considered intra- and inter-group variability. To verify whether the model was well adjusted to the data, a residual analysis was performed. Comparisons between groups within each time and between times within each group were performed considering orthogonal contrasts. The analyzes were implemented in the SAS program version 9.4. Differences were considered significant at P <0.05.

## 3 Results

**Table 1** presents the means ± standard deviation (SD), while **Tables 2**, **3** present intragroup (**Table 2**) and intergroup (**Table 3**) comparisons between the means of confidence intervals (CI) of the anthropometric characteristics and hemodynamic, hormonal and metabolic parameters. Anthropometric characteristics did not differ in intragroup (**Table 2**) or intergroup (**Table 3**). Regarding hemodynamic parameters, the period of 16 weeks (time factor) did not influence the values of heart rate (control group, P < 0.452) and mean arterial pressure (control group, P < 0.184). In turn, the aerobic physical training (training factor) reduced HR (MICT, P <.0.001; HIIT, P < 0.001). Both time and training factors had different influences on the metabolic parameters. The time factor reduced VO2peak and increased the testosterone (P < 0.035) and cholesterol (P < 0.009) levels in the control group, while the training factor increased VO2peak and decreased the testosterone level in the MICT (P < 0.001; P < 0.037, respectively) and HIIT (P < 0.001; P < 0.012, respectively) groups. Finally, both training protocols showed similar effects on all metabolic parameters evaluated, including VO2peak (P < 0.659).

**Table 1:** The descriptive table of characteristics and hemodynamic, hormonal and metabolic parameters obtained before and after the 16-wk physical training (MICT and HIIT groups) or observation period without training (Control group).

	Control (N=25)		MICT (N=25)		HIIT (N=25)	
	Before	After	Before	After	Before	After
Characteristics						
Age, years	29 ± 5	–	29 ± 5	–	29 ± 4	–
Height, m	1.61 ± 0.07	–	1.62 ± 0.06	–	1.64 ± 0.07	–
Weight, kg	76 ± 15	76 ± 16	73 ± 16	72 ± 16	76 ± 16	75 ± 16
BMI, kg/m^2^	29.2 ± 5.4	29.3 ± 5.4	27.7 ± 5.7	27.5± 7.5	27.8 ± 4.2	27.8 ± 4.2
Baseline Cardiovascular Values						
HR (bpm)	69 ± 11	69 ± 10	70 ± 12	66 ± 10	70 ± 10	66 ± 11
SBP (mmHg)	102 ± 11	108 ± 14	106 ± 12	101 ± 12	102 ± 9	103 ± 14
DBP (mmHg)	70 ± 10	71 ± 9	70 ± 10	69 ± 10	68 ± 8	67 ± 11
MAP (mmHg)	81 ± 10	83 ± 10	82 ± 9	80 ± 10	79 ± 7	79 ± 11
Hormonal and Metabolic Values						
VO_2peak_, mL/min/kg	33.7 ± 5.3	32.3 ± 5.1	32.5 ± 4.7	36.2 ± 5.0 *^c^*	33.6 ± 3.7	36.9 ± 4.3
Testosterone, ng/dL	86.7 ± 36.9	94.1 ± 42.3	104.7 ± 36.9	87.8 ± 33.3	98.8 ± 41.2	78.6 ± 51.8
Glucose, mg/dL	83.5 ± 7.1	81.8 ± 10.1	82.6 ± 8.4	81.6 ± 6.8	81.9 ± 8.8	81.4 ± 6.6
Insulin, μIU/mL	13.2 ± 9.2	12.8 ± 10.6	12.8 ± 8.1	11.7 ± 8.2	12.1 ± 5.2	11.8 ± 6.3
HOMA-IR	2.69 ± 1.9	2.56 ± 2.3	2.60 ± 1.7	2.41 ± 1.7	2.52 ± 1	2.34 ± 1.3
Triglyceride, mg/dL	117.7 ± 57.5	106 ± 62.9	125 ± 110.3	127.3 ± 91.1	102.4 ± 56.5	110.3 ± 63.6
Cholesterol, mg/dL	189.7 ± 35.4	177.7 ± 24.6	183.4 ± 28	173.2 ± 29.1	180.9 ± 31.1	175 ± 28
HDL, mg/dL	49.6 ± 12.4	47.7 ± 10.6	45.8 ± 8.4	44.7 ± 9.7	48.3 ± 10.6	46.2 ± 9.9
LDL, mg/dL	116.5 ± 33.3	108.6 ± 28.2	109.3 ± 21.8	103.4 ± 24.3	114.3 ± 19.3	107.4 ± 23.7

Values are expressed as means ± SD, standard deviation. MICT, moderate-intensity continuous training; HIIT, high-intensity interval training; BMI, body mass index; HR, heart rate; bpm, beats per minute; SBP, systolic blood pressure; DBP, diastolic blood pressure; MBP, mean blood pressure; VO_2peak_, oxygen uptake at peak exercise; HOMA-IR, homeostatic model assessment for insulin resistance; HDL, high-density lipoprotein; LDL, low-density lipoprotein.

**Table 2:** Intragroup comparison of baseline hemodynamic, hormonal and metabolic parameters obtained before and after the 16-wk of physical training.

	Control (N=25)		MICT (N=25)		HIIT (N=25)	
	Estimation of difference (CI 95%)	*P value*	Estimation of difference (CI 95%)	*P value*	Estimation of difference (CI 95%)	*P value*
Baseline hemodynamic parameters						
HR (bpm)	-0.64 (-2.33; 1.06)	.452	4.48 (2.79; 6.17)	< 0.001	4.52 (2.83; 6.22)	< 0.001
SBP (mmHg)	-5.52 (-10.41; -0.62)	.028	4.56 (-0.33; 9.45)	.067	-1.40 (-6.29; 3.49)	.570
DBP (mmHg)	-0.84 (-4.44; 2.76)	.640	0.80 (-2.80; 4.40)	.660	-0.24 (-3.84; 3.36)	.895
MBP (mmHg)	-2.36 (-5.87; 1.15)	.184	2.04 (-1.47; 5.55)	.251	-0.60 (-4.11; 2.91)	.735
Hormonal and Metabolic Values						
VO_2peak_, mL/min/kg	1.33 (0.06; 2.6)	.040	-3.79 (-5.05; -2.52)	< 0.001	-3.07 (-4.34; -1.81)	< 0.001
BMI, kg/m^2^	-0.14 (-0.50; 0.21)	.432	0.16 (-0,19; 0.52)	.368	0.04 (-0,32; 0.39)	.826
Testosterone, ng/dL	-7.36 (-23.01; -8.29)	.035	16.72 (1.07; 32.37)	.037	20.28 (4.63; 35.93)	.012
Glucose, mg/dL	1.66 (-1.0; -4.29)	.023	1.01 (-1.73; 3.75)	.465	0.53 (-2.21; 3.27)	.700
Insulin, μIU/mL	0.22 (-2.34; 2.78)	.862	1.07 (-1.49; 3.63)	.406	0.30 (-2.26; 2.86)	.814
HOMA-IR	0.15 (-0.37; 0.67)	.561	0.20 (-0.32; 0.72)	.444	0.18 (-0.34; 0.69)	.491
Triglyceride, mg/dL	11.72 (-9.54; 32.98)	.276	0.40 (-20.86; 21.66)	.970	2.16 (-19.10; 23.42)	.840
Cholesterol, mg/dL	12.0 (3.0; 21.0)	.009	10.24 (-1.24; 20.24)	.076	6.0 (-3.0; 15.0)	.188
HDL, mg/dL	1.90 (-1.40; 5.21)	.255	1.12 (-2.20; 4.42)	.503	2.16 (-1.15; 5.47)	.197
LDL, mg/dL	7.84 (-1.92; 17.6)	.114	5.92 (-3.84; 15.68)	.231	6.96 (-2.80; 16.72)	.160

Data are presented as means of the confidence intervals (CI) with their respective minimum and maximum values. Statistical analysis was performed by calculating the difference between subtracting the values obtained before and after the 16-week experimental protocol (estimation of difference= before - after). MICT, moderate-intensity continuous training; HIIT, high-intensity interval training; HR, heart rate; bpm, beats per minute; SBP, systolic blood pressure; DBP, diastolic blood pressure; MBP, mean blood pressure; VO_2peak_, oxygen uptake at peak exercise; BMI, body mass index; HOMA-IR, homeostatic model assessment for insulin resistance; HDL, high-density lipoprotein; LDL, low-density lipoprotein.

**Table 3:** Intergroup comparison of baseline hemodynamic, hormonal and metabolic parameters obtained after the 16-wk of physical training.

	Control *vs.* MICT		Control *vs.* HIIT		MICT *vs.* HIIT	
	Estimation of difference (CI 95%)	*P value*	Estimation of difference (CI 95%)	*P value*	Estimation of difference (CI 95%)	*P value*
**Baseline hemodynamic parameters**						
HR (bpm)	4.12 (-1.60; 9.84)	.155	4.16 (-1.56; 9.88)	.151	0.04 (-5.68; 5.76)	.989
SBP (mmHg)	7.00 (0.21; 13.79)	.044	4.92 (-1.87; 11.71)	.153	-2.08 (-8.87; 4.71)	.544
DBP (mmHg)	1.88 (-3.48; 7.24)	.487	3.32 (-2.04; 8.68)	.221	1.44 (-3.92; 6.80)	.594
MBP (mmHg)	3.56 (-1.86; 8.98)	.195	3.88 (-1.54; 9.30)	.158	0.32 (-5.10; 5.74)	.908
**Hormonal and Metabolic Values**						
VO_2peak_, mL/min/kg	-3.95 (-6.63; -1.27)	.005	-4.54 (-7.23; -1.86)	0.001	-0.60 (-3.28; 2.09)	.659
BMI, kg/m^2^	1.84 (-1.07; 4.75)	.211	1.57 (-1.34; 4.48)	.285	-0.27 (-3.18; 2.64)	.854
Testosterone, ng/dL	6.20 (-16.74; 29.14)	.591	15.52 (-7.42; 38.46)	.182	9.32 (-13.62; 32.26)	.421
Glucose, mg/dL	0.20 (-4.35; 4.75)	.930	0.44 (-4.11; 4.99)	.848	0.24 (-4.31; 4.79)	.917
Insulin, μIU/mL	1.30 (-3.26; 5.86)	.571	1.19 (-3.37; 5.75)	.605	-0.11 (-4.67; 4.45)	.961
HOMA-IR	1.14 (-0.83; 1.11)	.768	0.22 (-0.75; 1.19)	.652	0.08 (-0.89; 1.05)	.876
Triglyceride, mg/dL	-4.76 (-42.64; 33.12)	.803	-1.36 (-39.24; 36.52)	.943	3.4 (-34.48; 41.28)	.859
Cholesterol, mg/dL	4.56 (-12.08; 21.20)	.587	2.84 (-13.80; 19.48)	.735	-1.72 (-18.36; 14.92)	.837
HDL, mg/dL	3.03 (-2.79; 8.85)	.303	1.56 (-4.26; 7.38)	.596	-1.47 (-7.29; 4.35)	.616
LDL, mg/dL	5.28 (-9.11; 19.67)	.467	1.28 (-13.11; 15.67)	.860	-4.00 (-18.39; 10.38)	.581

Data are presented as means of the confidence intervals (CI) with their respective minimum and maximum values. MICT, moderate-intensity continuous training; HIIT, high-intensity interval training; HR, heart rate; bpm, beats per minute; SBP, systolic blood pressure; DBP, diastolic blood pressure; MBP, mean blood pressure; VO_2peak_, oxygen uptake at peak exercise; BMI, body mass index; HOMA-IR, homeostatic model assessment for insulin resistance; HDL, high-density lipoprotein; LDL, low-density lipoprotein.

**Table 4** presents the means ± SD, while **Tables 5**, **6** present intragroup (**Table 5**) and intergroup (**Table 6**) comparisons between the means of CI of all autonomic parameters evaluated. The analysis of HRV using linear methods (time domain and frequency domain) did not show any influence of time and physical training (MICT and HIIT) on cardiac autonomic parameters. In contrast, the symbolic analysis (non-linear method) showed an increase in 2UV% oscillations, which represents vagal modulation, in both trained groups (MICT, P < 0.036; HIIT, P < 0.017). **Tables 5**, **6** also present the results of BPV (linear analysis) and BRS, and the results showed that the time and physical training factors did not influence the evaluated parameters.

**Table 4:** The descriptive table of cardiovascular autonomic parameters obtained before and after the 16-wk of physical training (MICT and HIIT groups) or observation period without training (Control group).

	Control (N=25)		MICT (N=25)		HIIT (N=25)	
	Before	After	Before	After	Before	After
HRV - Time domain						
RRi, ms	866 ± 119	865 ± 115	829 ± 114	909 ± 94	823 ± 110	908 ± 126
SD	56 ± 31	57 ± 26	55 ± 23	56 ± 22	54 ± 26	62 ± 37
RMSSD, ms	54 ± 43	52 ± 33	52 ± 32	54 ± 27	48 ± 34	58 ± 45
HRV – Frequency domain						
Variance, ms^2^	2495 ± 2783	2906 ± 2507	2827 ± 2445	2990 ± 2352	2245 ± 2156	2515 ± 2521
LF, ms^2^	713 ± 741	851 ± 683	771 ± 808	737 ± 561	657 ± 710	620 ± 643
HF, ms^2^	1152 ± 1662	1295 ± 1525	1375 ± 1478	1208 ± 1164	791 ± 1052	888 ± 1141
LF, nu	45 ± 14	46 ± 18	42 ± 19	43 ± 14	48 ± 14	44 ± 13
HF, nu	55 ± 14	54 ± 18	58 ± 19	57 ± 14	52 ± 14	56 ± 13
LF/HF Ratio	0,96 ± 0.53	0.99 ± 0.57	0.98 ± 0.84	0.85 ± 0.49	0.96 ± 0.41	0.89 ± 0.53
HRV - Symbolic analysis						
0V %	21.9 ± 12	23.2 ± 15	23.2 ± 12	17.9 ± 11	26.1 ± 13	20.4 ± 9.6
2UV %	22.1 ± 12	20 ± 12	18.7 ± 7.5	22.6 ± 8.9	19.1 ± 9.7	23.5 ± 12
BPV – Frequency domain						
Variance, mmHg^2^	19 ± 8.7	20.7 ± 8.6	19.5 ± 9.4	20 ± 12.5	23.8 ± 12.1	20.2 ± 10.4
LF, mmHg^2^	4.9 ± 2.3	5.5 ± 2.2	4.9 ± 2.7	5.6 ± 3.4	6.6 ± 3.1	6.2 ± 3.7
BRS						
Ramp numbers	87 ± 46	82 ± 41	83 ± 48	85 ± 42	86 ± 38	78 ± 35
BEI	0.59 ± 0.14	0.58 ± 0.19	0.55 ± 0.19	0.62 ± 0.12	0.63 ± 0.12	0.6 ± 0.14
UP, ms/mmHg	15.7 ± 10.6	15.4 ± 8.3	14 ± 8.5	16 ± 8.1	13.5 ± 6.6	16 ± 8.3
DOWN, ms/mmHg	16.5 ± 9.4	16.6 ± 7.9	14.4 ± 7.5	16.6 ± 7.5	14.6 ± 6.9	17.4 ± 8.8
GAIN, ms/mmHg	16.2 ± 9.7	16.1 ± 7.9	14.3 ± 7.9	16.5 ± 7.4	14.1 ± 6.7	16.8 ± 8.6

Values are expressed as means ± SD, standard deviation. MICT, moderate-intensity continuous training; HIIT, high-intensity interval training; HRV, heart rate variability; RRi, R-R interval; ms, millisecond; SD, standard deviation; RMSSD, root mean square of successive normal sinus RR-interval; LF, low-frequency; HF, high-frequency; nu, normalized unit; 0V%, patterns with no variation (sympathetic modulation); 2UV, patterns with two unlike variation (vagal modulation); BPV, blood pressure variability; BEI, baroreflex effectiveness index.

**Table 5:** Intragroup comparison of autonomic parameters obtained before and after the 16-wk of physical training.

	Control (N=25)		MICT (N=25)		HIIT (N=25)	
	Estimation of difference (CI 95%)	*P value*	Estimation of difference (CI 95%)	*P value*	Estimation of difference (CI 95%)	*P value*
**HRV – Time domain**						
RRi, ms	0.73 (-47.96; 49.43)	.976	-76.64 (-125.34; -27.95)	0.003	-85.02 (-133.72; -36.33)	<0.001
SD, ms	-1.91 (-11.77; 7.94)	.700	-1.56 (-11.41; 8.30)	.754	-7.90 (-17.76; 1.95)	.114
RMSSD, ms	1.25 (-10.23; 12.73)	.829	-1.36 (-12.85; 10.11)	.813	-9.17 (-20.65; 2.31)	.116
**HRV – Frequency domain**						
Variance, ms^2^	-410.42 (-1161.18; 340.35)	.280	-163.50 (-914.27; 587.26)	.666	-270.28 (-1021.05; 480.48)	.475
LF, ms^2^	-138.18 (-386.52; 110.15)	.271	33.53 (-214,81; 281.87)	.789	37.23 (-211,11; 285.57)	.766
HF, ms^2^	-143.02 (-533.65; 247.61)	.468	166.32 (-224.31; 556.94)	.399	-97.17 (-487.80; 293.46)	.622
LF, nu	-1.34 (-7.87; 5.20)	.685	-1.03 (-7.57; 5.50)	.754	4.32 (-2.21; 10.86)	.191
HF, nu	1.34 (-5.20; 7.87)	.685	1.03 (-5.50; 7.57)	.754	-4.32 (-10.86; 2.21)	.191
LF/HF Ratio	-0.04 (-0.27; 0.19)	.734	0.13 (-0.11; 0.36)	.278	0.08 (-0.16; 0.31)	.519
**HRV – Symbolic analysis**						
0V %	-1.33 (-7.04; 4.38)	.643	5.27 (-0.44; 10.98)	.070	5.71 (0.004; 11.42)	.050
2UV %	2.07 (-1.53; 5.66)	.255	-3.84 (-7.44; -0.25)	.036	-4.39 (-7.98; -0.79)	.017
**BPV – Frequency domain**						
Variance, mmHg^2^	-1.64 (-6.27; 3.0)	.484	-0.61 (-5.24; 4.03)	.795	3.65 (-0.99; 8.28)	.121
LF, mmHg^2^	-0.61 (-1.86; 0.65)	.337	-0.70 (-1.96; 0.55)	.267	0.40 (-0.85; 1.66)	.553
**BRS**						
Ramp numbers	4.95 (-11.19; 21.09)	.543	-1.40 (-17.54; 14.74)	.863	7.95 (-8.19; 24.09)	.330
BEI	0.12 (-0.06; 0.08)	.739	-0.07 (-0,14; 0.0004)	.050	0.03 (-0,04; 0.10)	.429
UP, ms/mmHg	0.35 (-3.79; 4.49)	.867	-2.03 (-6.17; 2.11)	.332	-2.47 (-6.61; 1.67)	.238
DOWN, ms/mmHg	-0.09 (-3.40; 3.22)	.958	-2.20 (-5.51; 1.12)	.190	-2.84 (-6.15; 0.48)	.092
GAIN, ms/mmHg	0.08 (-3.52; 3.68)	.967	-2.21 (-5.81; 1.39)	.226	-2.74 (-6.34; 0.86)	.134

Data are presented as means of the confidence intervals (CI) with their respective minimum and maximum values. Statistical analysis was performed by calculating the difference between subtracting the values obtained before and after the 16-week experimental protocol (estimation of difference= before - after). MICT, moderate-intensity continuous training; HIIT, high-intensity interval training; HRV, heart rate variability; RRi, R-R interval; ms, millisecond; SD, standard deviation; RMSSD, root mean square of successive normal sinus RR-interval; LF, low-frequency; HF, high-frequency; nu, normalized unit; 0V%, patterns with no variation (sympathetic modulation); 2UV, patterns with two unlike variation (vagal modulation); BPV, blood pressure variability; BEI, baroreflex effectiveness index.

**Table 6:** Intergroup comparison of autonomic parameters obtained after the 16-wk of physical training.

	Control *vs.* MICT		Control *vs.* HIIT		MICT *vs.* HIIT	
	Estimation of difference (CI 95%)	*P value*	Estimation of difference (CI 95%)	*P value*	Estimation of difference (CI 95%)	*P value*
HRV – Time domain						
RRi, ms	-41.03 (-105.09; 23.02)	.206	-43.56 (-107.62; -20.50)	.180	-2.53 (-66.58; 61.53)	.936
SD, ms	1.22 (-14.58; 17.03)	.879	-4.56 (-20.37; 11.24)	.567	-5.79 (-21.59; 10.02)	.468
RMSSD, ms	-1.19 (-21.48; 19.10)	.907	-5.08 (-25.37; 15.21)	.619	-3.89 (-24.18; 16.40)	.703
HRV – Frequency domain						
Variance, ms^2^	-84.48 (-1476.25; 1307.28)	.904	390.34 (-1001.43; 1782.10)	.578	474.82 (-916.95; 1866.59)	.499
LF, ms^2^	113.52 (-278.60; 505.64)	.566	230.48 (-161.64; 622.60)	.245	116.96 (-275.16; 509.08)	.554
HF, ms^2^	87.00 (-677.65; 851.64)	.821	407.01 (-357.63; 1171.65)	.292	320.02 (-444.63; 1084.66)	.407
LF, nu	3.54 (-5.23; 12.31)	.424	2.10 (-6.67; 10.87)	.635	-1.44 (-10.21; 7.33)	.745
HF, nu	-3.54 (-12.31; 5.23)	.424	-2.10 (-10.87; 6.67)	.635	1.44 (-7.33; 10.21)	.745
LF/HF Ratio	0.15 (-0.18; 0.48)	.370	0.10 (-0.23; 0.42)	.560	-0.05 (-0.38; 0.28)	.752
HRV – Symbolic analysis						
0V %	5.29 (-1.59; 12.17)	.130	2.79 (-4.09; 9.67)	.421	-2.50 (-9.38; 4.38)	.471
2UV %	-2.52 (-8.49; 3.46)	.404	-3.46 (-9.43; 2.51)	.251	-0.94 (-6.92; 5.03)	.754
BPV – Spectral Analysis						
Variance, mmHg^2^	0.52 (-5.35; 6.40)	.859	0.48 (-5.40; 6.35)	.872	-0.05 (-5.92; 5.82)	.987
LF, mmHg^2^	-0.06 (-1.72; 1.59)	.939	-0.63 (-2.29; 1.03)	.450	-0.57 (-2.23; 1.09)	.497
BRS						
Ramp numbers	2.60 (-26.16; 20.96)	.827	3.79 (-19.78; 27.35)	.750	6.39 (-17.18; 29.95)	.591
BEI	-0.05 (-0.13; 0.04)	.290	-0.03 (-0.11; 0.06)	.528	0.02 (-0.07; 0.11)	.667
UP, ms/mmHg	-0.97 (-5.76; 3.81)	.687	-0.60 (-5.38; 4.18)	.803	0.37 (-4.41; 5.16)	.877
DOWN, ms/mmHg	-0.05 (-4.59; 4.48)	.982	-0.86 (-5.40; 3.68)	.707	-0.81 (-5.34; 3.73)	.724
GAIN, ms/mmHg	-0.46 (-5.02; 4.10)	.841	-0.74 (-5.29; 3.82)	.748	-0.28 (-4.83; 4.28)	.904

Data are presented as means of the confidence intervals (CI) with their respective minimum and maximum values.

MICT, moderate-intensity continuous training; HIIT, high-intensity interval training; HRV, heart rate variability; RRi, R-R interval; ms, millisecond; SD, standard deviation; RMSSD, root mean square of successive normal sinus RR-interval; LF, low-frequency; HF, high-frequency; nu, normalized unit; 0V%, patterns with no variation (sympathetic modulation); 2UV, patterns with two unlike variation (vagal modulation); BPV, blood pressure variability; BEI, baroreflex effectiveness index.

**Figure 2:**
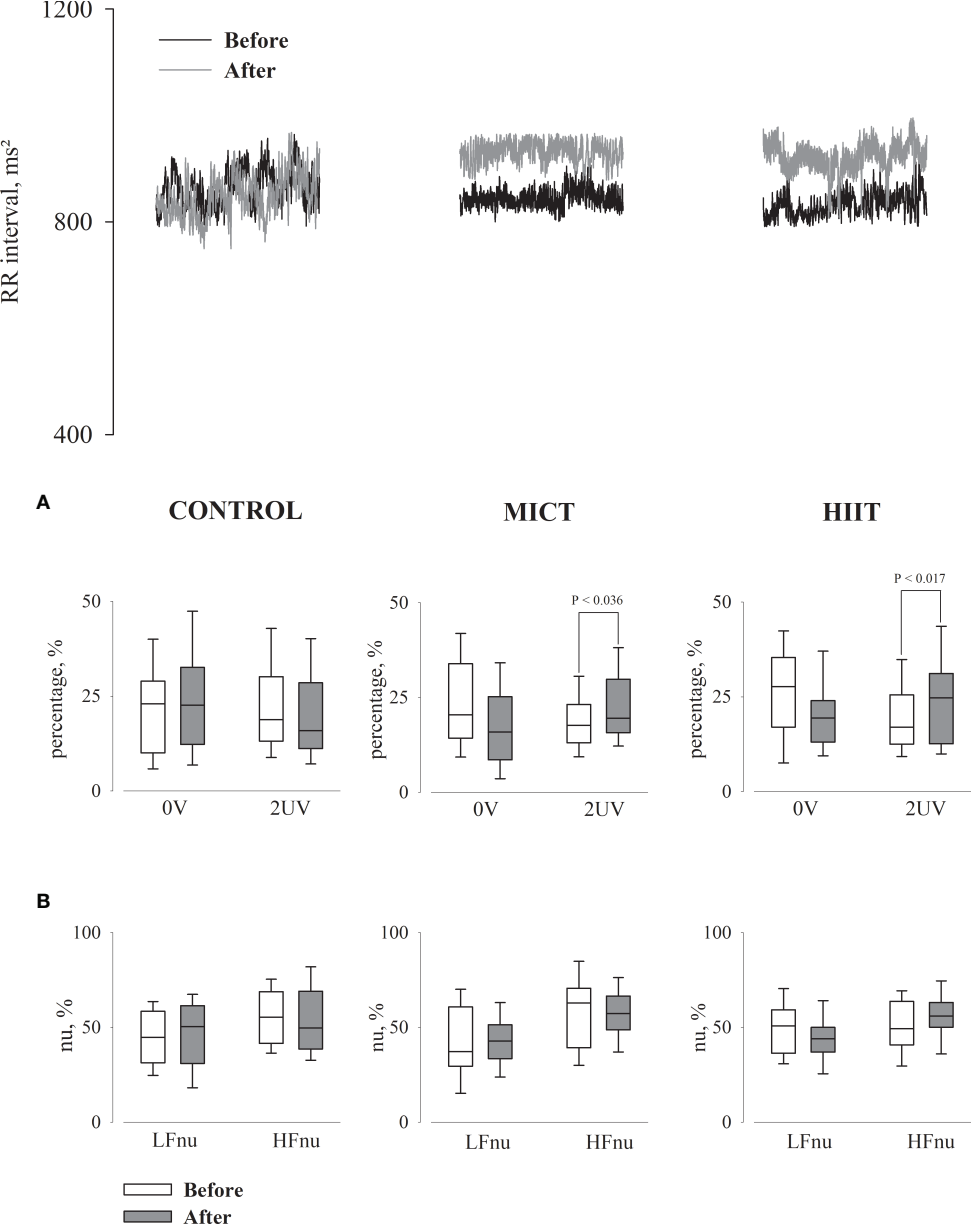
Cardiac interval segments obtained during rest in supine position for all groups, before and after 16 weeks of the intervention. **(A)** Graphs showing the symbolic analysis; 0V, patterns with no variation (sympathetic modulation); 2UV, patterns with two unlike variation (vagal modulation). **(B)** Graphs showing the spectral analysis in normalized units (nu); LF, low frequency band (sympathetic modulation); HF, high frequency band (vagal modulation).

**Figure 2** shows a representative record of the RRi segments of each group before and after 16 weeks of aerobic physical training. It also shows the results of the HRV analysis performed by a non-linear method (symbolic analysis, **Figure 2A**) and a linear method (spectral analysis, **Figure 2B**) using Fast Fourier Transform (FFT). In most parameters evaluated, the results were similar between the two methods. Only in the intragroup comparison, the symbolic analysis showed an increase in HRV corresponding to vagal modulation in the MICT and HIIT groups, characterized by an increase in 2UV variations.

## 4 Discussion

Our results showed that after a 16-week training period, MICT and HIIT groups presented with higher cardiorespiratory fitness than the control, characterized by an increase in VO_2peak_ and a decrease in baseline HR. Both models of physical training reduced testosterone levels, while the control group showed an increase. Metabolic parameters were similar between groups, and these observations may be associated with the maintenance of anthropometric parameters after the 16 weeks. All the participants were instructed to maintain a normal diet, and maintenance of anthropometric parameters was expected. However, we expected that both exercise training protocols would increase insulin sensitivity, which did not occur.

Before and after MICT and HIIT, the assessment of BRS and analysis of cardiovascular autonomic modulation using linear methods did show any changes. In this case, MICT and HIIT showed similar cardiovascular autonomic modulation results. In turn, the analysis of HRV through symbolic analysis (non-linear method) showed an increase in vagal modulation only in the HIIT group. In contrast, the comparison between groups showed no differences after 16 weeks. These results contradict the findings of other studies, which have shown that HIIT is more effective than MICT, particularly for some parameters, such as VO_2peak_ and HRV (11–13). However, the prescription of the two training protocols must be considered. In our study, we ensured that both training sessions had similar volumes, controlling not only the daily training time and intensity but also the number of heartbeats during each training session. Monitoring the number of heartbeats ensures that each group has a similar total number of heartbeats per training session (31). This prevents the HIIT group, which has a short training time, from having a greater number of heartbeats per session. During the high metabolic demand (2-min), the training HR reached 85%–90% of the reserve HR, after that, the treadmill’s speed and inclination returned to 65%–70% of the reserve HR, that is, the lowest metabolic demand intensity for 3-min. Thus, during the first week of training, especially for sedentary individuals, after reaching 85%–90% of the reserve HR, the exercise intensity decreases (65%–70%) and is not followed by a prompt reduction in HR, which corresponds to this lower intensity. Interestingly, when we consider the rigid pre-determined HIIT interval times, the total number of heartbeats obtained in the HIIT is often higher than the total number obtained in the MICT, even if the latter has a longer training duration. Thus, as mentioned in the methods section, we stipulated a flexible time range for HIIT, which was between 35 and 45 min. This range was used to allow all participants in the HIIT group to obtain similar numbers of total heartbeats compared to the participants in the MICT group. This enabled us to suggest that the most significant results obtained in other studies on the HIIT protocol can be partially explained by a greater volume of training, that is, the number of heartbeats (31). Another important aspect to consider is the variation in BP observed in this study during the physical training sessions, specifically in SBP. SBP reached higher values (≅ 145 mmHg) in the HIIT group than in the MICT group (≅ 130 mmHg). These higher SBP values in HIIT suggest greater endothelial shear stress, promoting prominent adaptations, such as the release of dilating factors derived from the endothelium (32–34). However, we found no statistical differences in resting BP between the training sessions and trained groups. Adherence to the two physical training protocols must also be considered. Despite the greater physical demand in the execution of HIIT, dropouts were slightly lower (N=10) in the HIIT group when compared to those in the MICT group (N=12).

Regardless of the HRV, BPV, and BRS analysis methods, the clinical relevance of these parameters is widely known as an important predictor of cardiac morbidity and mortality and is often used to investigate autonomic adaptations in cardiac regulation resulting from different diseases (9, 10, 25, 35–37). Moreover, these analysis can be influenced by different metabolic and hormonal variables, such as blood glucose levels, insulin levels, testosterone levels, ovarian hormone levels, obesity, and caffeine intake (15, 26, 38–40). The literature has shown beneficial effects of aerobic physical training on autonomic modulation in individuals with chronic diseases, evidenced by a reduction in sympathetic modulation and an increase in vagal modulation (7, 8, 15–17).

However, in the present study, the participants with PCOS showed modulatory autonomic values similar to those of participants without PCOS, based on other studies that used spectral analysis (linear analysis) as a tool (41, 42). This observation is important because autonomic modulation obtained by spectral analysis seemed intact in these groups. This might be associated with blood glucose and insulin levels, since there were no differences in these parameters between the groups, and the values were within the normal reference range.

In addition, cardiac autonomic modulation has been extensively studied, although there is no consensus among researchers and clinicians regarding the best methodology for the HRV analysis. Since the development of the first computational tools to analyze the physiological mechanisms involved in HRV, several linear and nonlinear methods have been used. The most widely used linear method is the spel analysis, both for clinical and experimental studies (25, 37). Among the various nonlinear methods, symbolic analysis has recently been highlighted (27, 28, 43). This analysis transforms three beats into symbol segments (0V, 1V, 2LV and 2UV) classifying them according to their pattern within the tachogram, created from the delta between the highest and the lowest RRi of the recorded HR (44). Each symbol represents an autonomic modulation response as follows: 0V represents sympathetic modulation, 1V represents both modulations, and 2UV represents vagal modulation (28). Our results suggest a greater sensitivity of symbolic analysis detecting changes in cardiac autonomic modulation, evidenced by the increase in vagal modulation (2UV%) in MICT and HIIT groups, however, in the intergroup comparison, no difference was observed. In this case, further studies are needed to assess whether there are advantages of symbolic (non-linear) analysis over spectral (linear) analysis.

The current study had some limitations: the mean BMI of all groups was classified as overweight, since the body fat percentage may influence cardiovascular autonomic modulation. The dosages of inflammatory markers and oxidative stress could have provided additional important information to differentiate the effects of the two training models. In addition, the application of a physical training period longer than 16 weeks could facilitate the discrimination of possible differences between the MICT and HIIT. However, it is important to note that these limitations do not invalidate the main findings of this study.

## 5 Conclusion

In conclusion, MICT and HIIT had similar effects on anthropometric, metabolic and hormonal parameters. Both increased cardiorespiratory fitness and reduced baseline HR and serum testosterone levels. They also did not differ in relation to the effects on cardiovascular autonomic modulation and BRS. In this case, the only finding was the increa in vagal autonomic modulation in both trained groups. However, this finding was only observed in the non-linear analysis. Thus, we did not find greater benefits of HIIT compared to MICT in women with PCOS for all parameters evaluated.

## Data availability statement

The raw data supporting the conclusions of this article will be made available by the authors, without undue reservation.

## Ethics statement

The studies involving human participants were reviewed and approved by Ethics Committee of the Ribeirão Preto Medical School’s Hospital (Protocol number 845.830/2014). The patients/participants provided their written informed consent to participate in this study.

## Author contributions

SP participated in the acquisition of data, analysis and interpretation of data, manuscript preparation for the original draft. VR participated in acquisition of data and project administration. JT participated in the interpretation of data and manuscript review for intellectual content. RR participated in the study design, conceptualization, and manuscript review. DG participated in the interpretation of data and statistical analysis. HS participated in the conception, design of the study, analysis and interpretation of data, and manuscript review. All authors have read and approved the final manuscript.

## Funding

This work was supported by the São Paulo Research Foundation – FAPESP (Process 2015/14031-0; 2018/08569-6; and 2018/17892-5).

## Acknowledgments

We would like to thank all the participants for their commthe itment and adherence to the training protocol.

## Conflict of interest

The authors declare that the research was conducted in the absence of any commercial or financial relationships that could be construed as a potential conflict of interest.

## Publisher’s note

All claims expressed in this article are solely those of the authors and do not necessarily represent those of their affiliated organizations, or those of the publisher, the editors and the reviewers. Any product that may be evaluated in this article, or claim that may be made by its manufacturer, is not guaranteed or endorsed by the publisher.
