# Supplementary material for: Cardiovascular autonomic modulation differences between moderate-intensity continuous and high-intensity interval aerobic training in women with PCOS: A randomized trial
Source: Front Endocrinol (Lausanne). 2022 Nov 25;13:1024844. doi: 10.3389/fendo.2022.1024844 (PMC9782449; doi:10.3389/fendo.2022.1024844)
Supplement: Supplementary file 1 [file Table_1.docx]

**Table 1 – Supplement.** Anthropometric and hemodynamic parameters obtained before and after the protocol for all three groups.

|  | **Control Group** | |  | **MICT Group** | |  | **HIIT Group** | |
| --- | --- | --- | --- | --- | --- | --- | --- | --- |
|  | Before  (n=25) | After  (n=25) |  | Before  (n=25) | After  (n=25) |  | Before  (n=25) | After  (n=25) |
| **Characteristics** |  |  |  |  |  |  |  |  |
| Age, years | 29 ± 5 | - |  | 29 ± 5 | - |  | 29 ± 4 | - |
| Height, m | 1.61 ± 0.07 | - |  | 1.62 ± 0.06 | - |  | 1.64 ± 0.07 | - |
| Weight, kg | 75 ± 15.1 | 76 ± 15.7 |  | 73 ± 16.3 | 72 ± 15.7 |  | 76 ± 15.7 | 75 ± 15.5 |
| BMI, kg/m^2^ | 29.2 ± 5.4 | 29.3 ± 5.4 |  | 27.7 ± 5.7 | 27.5± 7.5 |  | 27.8 ± 4.2 | 27.8 ± 4.2 |
| **Baseline Cardiovascular Values** | |  |  |  |  |  |  |  |
| HR (bpm) | 69 ± 11 | 69 ± 10 |  | 70 ± 12 | 66 ± 10 |  | 70 ± 10 | 66 ± 11 |
| SBP (mmHg) | 102 ± 11 | 108 ± 14 |  | 106 ± 12 | 101 ± 12 |  | 102 ± 8.7 | 103 ± 14 |
| DBP (mmHg) | 70 ± 10 | 71 ± 9 |  | 70 ± 10 | 69 ± 10 |  | 68 ± 8 | 67 ± 11 |
| MAP (mmHg) | 81 ± 9 | 83 ± 10 |  | 82 ± 9 | 80 ± 10 |  | 79 ± 7 | 79 ± 11 |
| **Metabolic Values** |  |  |  |  |  |  |  |  |
| VO_2peak_, mL/min/kg | 33.7 ± 5.3 | 32.3 ± 5.1 |  | 32.5 ± 4.7 | 36.2 ± 5.0 |  | 33.6 ± 3.7 | 36.9 ± 4.3 |
| Testosterone, ng/dL | 86.7 ± 36.9 | 94.1 ± 42.3 |  | 104.7 ± 36.9 | 87.8 ± 33.3 |  | 98.8 ± 41.2 | 78.6 ± 51.8 |
| Glucose, mg/dL | 83.5 ± 7.1 | 81.8 ± 10.1 |  | 82.6 ± 8.4 | 81.6 ± 6.8 |  | 81.9 ± 8.8 | 81.4 ± 6.6 |
| Insulin, μIU/mL | 13.2 ± 9.2 | 12.8 ± 10.6 |  | 12.8 ± 8.1 | 11.7 ± 8.2 |  | 12.1 ± 5.2 | 11.8 ± 6.3 |
| HOMA-IR | 2.69 ± 1.9 | 2.56 ± 2.3 |  | 2.60 ± 1.7 | 2.41 ± 1.7 |  | 2.52 ± 1 | 2.34 ± 1.3 |
| Triglyceride, mg/dL | 117.7 ± 57.5 | 106 ± 62.9 |  | 125 ± 110.3 | 127.3 ± 91.1 |  | 102.4 ± 56.5 | 110.3 ± 63.6 |
| Cholesterol, mg/dL | 189.7 ± 35.4 | 177.7 ± 24.6 |  | 183.4 ± 28 | 173.2 ± 29.1 |  | 180.9 ± 31.1 | 175 ± 28 |
| HDL, mg/dL | 49.6 ± 12.4 | 47.7 ± 10.6 |  | 45.8 ± 8.4 | 44.7 ± 9.7 |  | 48.3 ± 10.6 | 46.2 ± 9.9 |
| LDL, mg/dL | 116.5 ± 33.3 | 108.6 ± 28.2 |  | 109.3 ± 21.8 | 103.4 ± 24.3 |  | 114.3 ± 19.3 | 107.4 ± 23.7 |

Values expressed as means ± SD, standard deviation. MICT, moderate intensity aerobic training; HIIT, high intensity interval training; time factor, 16-weeks considered between evaluations; BMI, body mass index; HR, heart rate; SBP, systolic blood pressure; DBP, diastolic blood pressure; MBP, mean blood pressure.
